# Targeting Cullin-RING E3 Ligases for Radiosensitization: From NEDDylation Inhibition to PROTACs

**DOI:** 10.3389/fonc.2020.01517

**Published:** 2020-08-21

**Authors:** Shuhua Zheng, Wensi Tao

**Affiliations:** ^1^College of Osteopathic Medicine, Nova Southeastern University, Fort Lauderdale, FL, United States; ^2^Department of Radiation Oncology, University of Miami-Miller School of Medicine, Coral Gables, FL, United States

**Keywords:** NEDDylation, EGFR, PROTAC, cullin-RING E3 ligase, MLN4924

## Abstract

As a dynamic regulator for short-lived protein degradation and turnover, the ubiquitin-proteasome system (UPS) plays important roles in various biological processes, including response to cellular stress, regulation of cell cycle progression, and carcinogenesis. Over the past decade, research on targeting the cullin-RING (really interesting new gene) E3 ligases (CRLs) in the UPS has gained great momentum with the entry of late-phase clinical trials of its novel inhibitors MLN4924 (pevonedistat) and TAS4464. Several preclinical studies have demonstrated the efficacy of MLN4924 as a radiosensitizer, mainly due to its unique cytotoxic properties, including induction of DNA damage response, cell cycle checkpoints dysregulation, and inhibition of NF-κB and mTOR pathways. Recently, the PROteolysis TArgeting Chimeras (PROTACs) technology was developed to recruit the target proteins for CRL-mediated polyubiquitination, overcoming the resistance that develops inevitably with traditional targeted therapies. First-in-class cell-permeable PROTACs against critical radioresistance conferring proteins, including the epidermal growth factor receptor (EGFR), androgen receptor (AR) and estrogen receptor (ER), cyclin-dependent kinases (CDKs), MAP kinase kinase 1 (MEK1), and MEK2, have emerged in the past 5 years. In this review article, we will summarize the most important research findings of targeting CRLs for radiosensitization.

## Introduction

Over 60% of cancer patients undergo radiotherapy (RT) during their course of illness, with an estimated 40% contribution toward curative cancer treatment (1, 2). While RT is an essential element for curative, adjuvant, and palliative treatment of a range of human malignancies, a key challenge in RT is to maximize radiation doses to the tumor mass while sparing the surrounding healthy tissue (1). To that end, various approaches combining RT with chemotherapies as radiosensitizers have been explored, which led to improvements in tumor response and higher overall survival (OS) rates (3). Despite a clear success, the favorable clinical outcome of chemoradiotherapy still comes at the sacrifice of increased toxicity in many clinical contexts, mainly due to the limited specificity of conventional chemotherapies (4). In the past two decades, several clinical trials have been conducted to test combining RT with targeted therapies against radioresistance conferring proteins such as epidermal growth factor receptor (EGFR), histone deacetylase (HDAC) and the B-rapidly accelerated fibrosarcoma (BRAF), aiming to develop combined-modality treatment regimens with fewer side effects (5–7). Clinical studies consistently suggested increased efficacy and improved survival rates of these new strategies, highlighting the clinical importance of using targeted agents as radiosensitizers (4). However, cancer cells will inevitably develop resistance toward these targeted therapies, leading to disease progression and relapse (8). Therefore, there is an urgent need to develop new strategies for radiosensitization.

In the past decade, targeting the activities of cullin-RING (really interesting new gene) E3 ligases (CRLs) in the ubiquitin-proteasome system (UPS) has gained considerable momentum for cancer treatment with the entry of several late-phase clinical trials of its first-in-class inhibitor MLN4924 (pevonedistat) (9). As early as the 1990s, the implication of targeting CRL for radiosensitization was suggested when its key component RING box protein 2 (Rbx2, a.k.a., SAG, ROC2) was identified as a redox inducible antioxidant protein (10). In recent years, studies showed that CRLs carry out the turnover of vital proteins involved in DNA damage response (DDR), as well as those in cell signaling pathways that are critical for radiosensitization (9, 11). Furthermore, in the past 5 years, the development of cell-permeable PROteolysis TArgeting Chimeras (PROTACs), which can selectively recruit radioresistance conferring proteins for CRL-mediated polyubiquitination, paved new methods in developing radiosensitizers that are less likely to develop chemoresistance (12). As such, it is crucial to systematically overview the mechanism of actions of CRL inhibitors for radiosensitization.

In this review article, we will summarize major strategies targeting CRLs and evaluate their potential as radiosensitizers based on the revised framework of the Steel hypothesis, originally described by George Steel in the 1970s (4). The revised hypothesis describes the scenario whereby combined-modality of targeted therapies and RT can improve the therapeutic outcomes by five mechanisms: (1) spatial cooperation, (2) temporal modulation, (3) biological cooperation, (4) cytotoxic enhancement, and (5) normal tissue protection (4).

## A Glimpse of Traditional Radiosensitizers

Typical RT involves ionizing radiation (IR), which uses high-energy photon radiation, such as X-rays and gamma (γ) rays, and particle radiation, such as electron (e), carbon ion and proton (13, 14). The IR exerts cytotoxic effects via direct DNA damage, or indirectly via generation of free radicals, particularly reactive oxygen species (ROS) (15–17). Therefore, the radiosensitivity of cancer cells can be influenced by biological factors that regulate DNA damage repair, oxygen perfusion levels, and cell cycle stage (16). Traditional radiosensitizers target these underlying parameters for radiosensitization.

Platinum analogs, 5-fluorouracil (5-FU), and taxanes are the most common clinically used radiosensitizers. Platinum analogs, such as cisplatin and oxaliplatin, can bind to DNA and produce DNA-DNA crosslinking, which will lead to cell cycle arrest and exacerbating the radiation-induced DNA damage (18). Meanwhile, 5-FU, capecitabine (a 5-FU oral prodrug), and gemcitabine act as pseudo-substrates, incorporation of these nucleoside analogs can dysregulate cell cycle checkpoint in the S phase, disabling DNA damage repair machinery in cancer cells upon IR administration (19, 20). On the other hand, taxanes, such as paclitaxel and docetaxel, synchronize tumor cells at cell cycle G2-M phase and trigger chromosomal missegregation (21, 22). Meanwhile, tumors in the hypoxic microenvironment (low pO_2_) are more radioresistant than those well-oxygenated (13). At the presence of oxygen, RT-induced DNA damages will be “fixed” via the formation of peroxyl radicals in DNA that had been insulted by free radicals (23). The oxygen mimics such as nitroimidazole derivatives (i.e., pimonidazole and nimorazole), and hypoxia-specific toxins were investigated in clinical trials as radiosensitizers (24, 25). Wang et al. (16) provided a comprehensive review on the recent development of radiosensitizers based on these principles.

The therapeutic potential of radiosensitizer is largely determined by the enhanced efficacy and selectivity against cancer cells but not normal tissue. However, traditional radiosensitizers are also chemotherapeutic drugs, which can cause prominent side effects. For example, cisplatin can cause intolerable nausea, vomiting, hearing loss, and kidney damage (26). Targeted therapies, such as MLN4924 and PROTACs are highly selective and would have fewer side effects. In fact, clinical trials of MLN4924 showed that this compound is well-tolerated (27).

## Targeting the Ubiquitin-Proteasome System (UPS)

**Figure 1:**
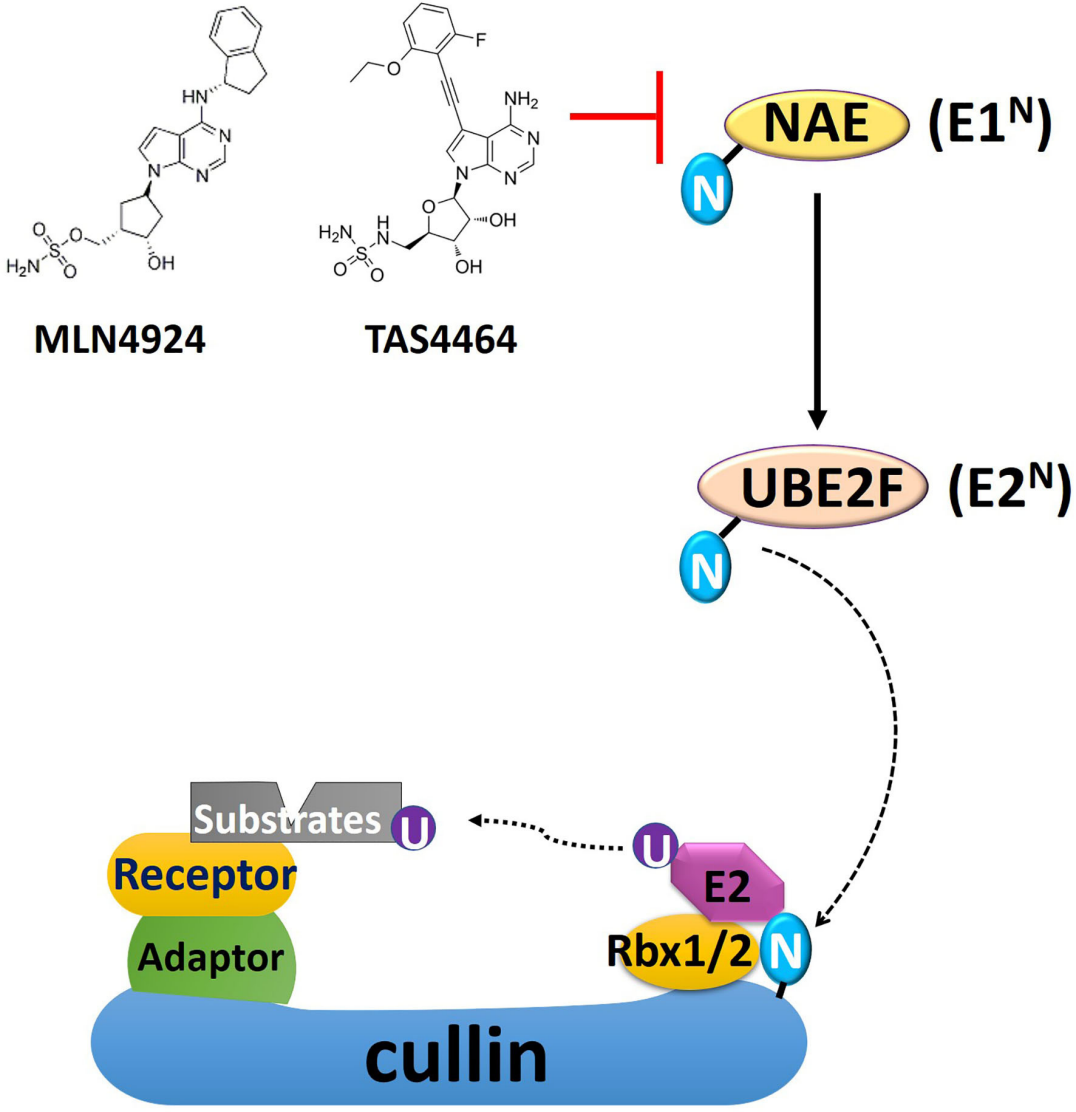
A schematic overview of cullin-RING E3 ligase (CRL) and NEDD8 conjugation. Conjugation of NEDD8 to the scaffold cullin protein in the CRL is carried out in three enzymatic steps involving NEDD8-activating enzyme (NAE, E1^N^), UBC12, and UBE2F (E2^N^). The substrate receptor protein, docked in the CRL complex by binding to the adaptor protein, recruits substrates for ubiquitin conjugation. MLN4924 and TAS4464 are specific NAE inhibitors, prohibiting NEDD8 conjugation and thus inhibit CRL activity. The 2-D structure of MLN4924 and TAS4464 was derived from Yu et al. (34). N, NEDD8; U, ubiquitin; Rbx, RING box protein.

**Figure 2:**
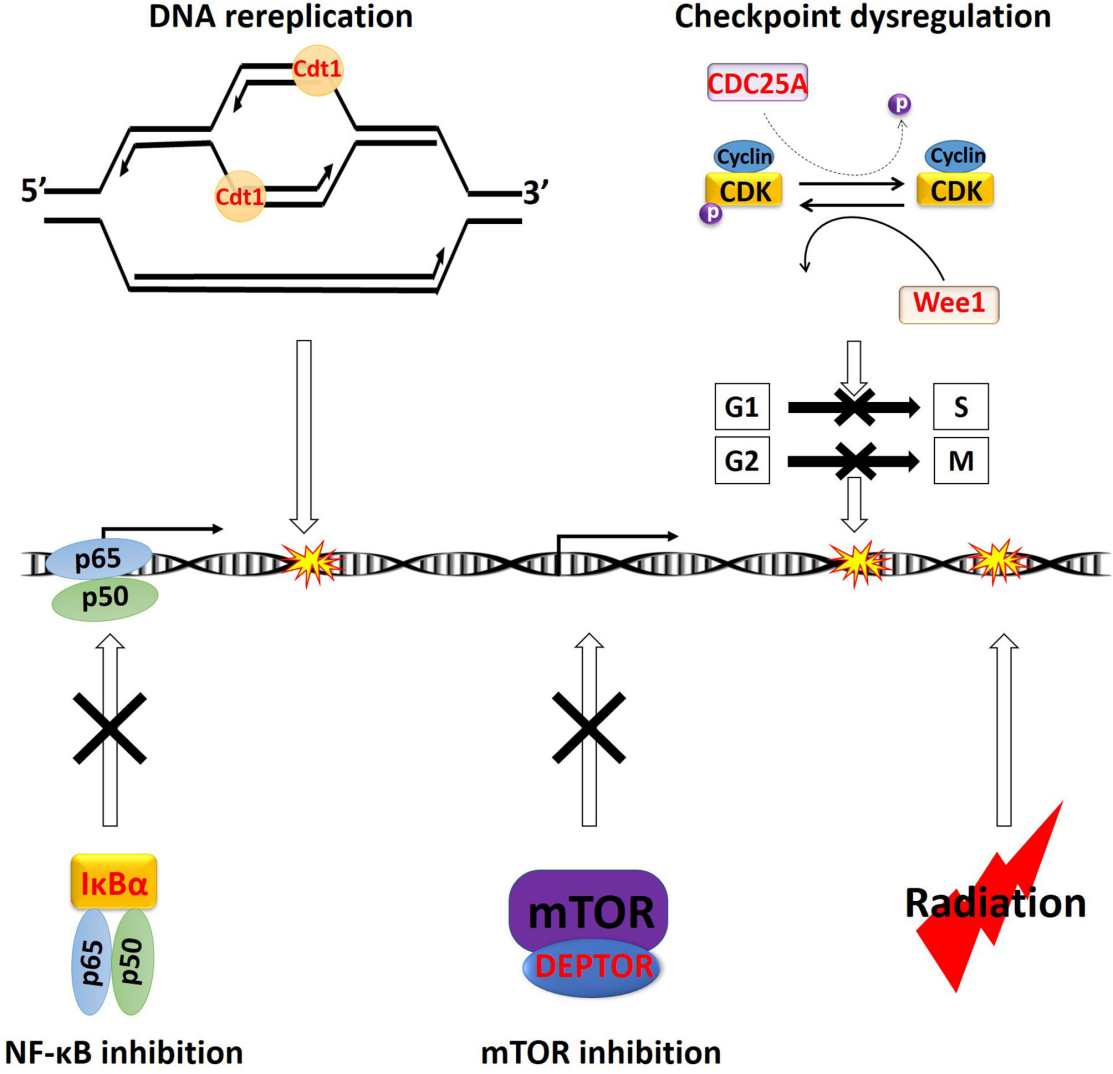
Overview of the major cytotoxic mechanism of NEDDylation inhibition. All the characters in “Red” color are CRL substrates. Accumulation of Cdt1 can trigger DNA rereplication, resulting in DNA damages. Stabilization of CDC25A can potentially cause dysregulation of both early (G1 phase to S phase) and late (G2 phase to M phase) cell cycle checkpoints (dash arrow). Meanwhile, the accumulation of Wee1, p21, and p27 will trigger cell cycle arrest at the G1 or G2 phase. Stabilization of IκBα will lead to sequestration NF-κB p65 and p50 heterodimer in the cytosol, leading to inhibition of its transcriptional activities. Stabilization of DEPTOR can directly inhibit mTOR. Ionizing radiation also triggers cell death via induction of DNA damages. p, phosphor group.

Cellular protein levels are tightly controlled by both protein synthesis and degradation. The ubiquitin-proteasome system (UPS), first characterized in the mid-20th century, is a dynamically regulated multi-enzyme process that earmarks substrate proteins for proteasomal-mediated degradation via polyubiquitination (28). Targeted inhibition of the UPS via direct eradication of the proteasome activities using bortezomib, carfilzomib, or ixazomib has been proven clinically effective for treating multiple myeloma (MM) (29). Several clinical trials also investigated the UPS inhibitors for their potential as radiosensitizers in the treatment of metastatic melanoma (Phase I), head and neck cancer (Phase I), and glioblastoma multiforme (GBM; Phase II) (30–32). However, unexpected earlier tumor progression as a result of EGFR stabilization has been reported with the combined administration of bortezomib and conventional radiochemotherapy in head and neck cancer (32, 33). Such suboptimal response is conceivable as proteasome inhibition indiscriminately stabilizes the substrates, including EGFR and other oncogenic proteins, limiting the clinical applications in targeting proteasome as a radiosensitizing strategy (32). Instead of directly inhibiting the proteasome, recent studies have employed alternative strategies such as targeting the UPS via inhibition of the upstream ubiquitin (Ub) conjugation events or directly recruiting specific substrate protein for polyubiquitination using PROteolysis TArgeting Chimeras (PROTACs) (Figures 1, 2).

## NEDDylation Inhibition

### NEDD8 Conjugation Pathway

The cullin-RING E3 ligases (CRLs) are responsible for polyubiquitination of about 20% of cellular proteins degraded via the UPS, most of which are critically involved in cell cycle progression, DDR, and oncogenic signaling cascades (9). The CRL complex's core structure is formed with a scaffold protein cullin bound with the RING-finger containing proteins (Rbx1 or Rbx2) at the C-terminus of cullin (35). This core complex will be joined by the adaptor protein, which binds the cullin's N-terminus domain to form a complete CRL complex (Figure 1) (35). Fully activation of CRLs requires conjugation of an Ub-like protein called neural precursor cell-expressed developmentally downregulated 8 (NEDD8) to near the C-terminus of the cullin in the CRL complex (11). Conjugation of NEDD8 to cullins is carried out in three enzymatic steps involving NEDD8-activating enzyme (NAE; E1), UBC12 and UBE2F (E2s), and E3s (Figure 1). NAE adenylates NEDD8 on its C-terminal glycine, forming a NEDD8-NAE complex via a covalent thiol-ester bond, and then transfers NEDD8 to the E2s via another thiol-ester bond (36, 37). NEDD8 E3 ligases execute the final step in conjugating NEDD8 to cullins, forming an isopeptide bond with the ε-amino group of a substrate lysyl residue (38, 39). NEDD8 conjugation facilitates the CRL structural remodeling that will juxtapose the substrate toward the catalytically active ubiquitylation site of CRL (40–42). The substrate receptor protein, docked in the CRL complex by binding to the adaptor protein, recruits substrates for Ub conjugation once CRL is fully functional (43) (Figure 1). So far, seven different types of human cullin proteins (CUL1, 2, 3, 4A, 4B, 5, 7) have been identified, and new members of the receptor and adaptor proteins are emerging (44). A more detailed overview of NEDD8 conjugation in CRLs has been summarized by Petroski and Deshaies in their review paper (45).

MLN4924 is an adenosine sulfamate analog that inhibits NEDDylation via the formation of an MLN4924-NEDD8 adduct, blocking the downstream NEDD8 conjugation cascade within a few hours after administration (46) (Figure 1). TAS4464 is another NAE inhibitor recently developed with more potent inhibitory effects and prolonged duration of target-binding compared with MLN4924 (47, 48). NAE inhibitors MLN4924 and TAS4464 targeting the process of NEDD8 conjugation have shown particularly promising results in several clinical trials (phase I/II/III) for cancer treatment (46). NEDDylation inhibition appears to have a unique profile of sensitivity toward various types of malignancies. So far, the primary identified cytotoxic mechanisms of MLN4924 include induction of DNA rereplication, senescence, dysregulation of cell cycle checkpoints control, as well as inhibition of mTOR and NF-κB pathways (Figure 2) (46, 49–55). Substrates of CRLs, such as Wee1, checkpoint kinase 1 (CHK1), p21, and cell division cycle 25A (CDC25A), are key components of double-strand breaks (DSBs) repair proteins (56–60) (Figure 2). Mainly, the degradation of cell cycle proteins Cdt1, p21, and Set8 is mediated by CRL4^Cdt2^, in which Cdt2 plays as a substrate recognition protein (61–63). Genotoxic insults trigger binding of Cdt2 on to the DNA sliding clamp—proliferating cell nuclear antigen (PCNA)—loading CRL4^Cdt2^ on to DNA for the substrate degradation (61–63). Set8 stabilization leads to lysine 20 of histone H4 (H4K20) hypermethylation, triggering transcriptional downregulation of histone with the resultant chromatin decompaction and DNA damage activation, as depicted elegantly by Abbas et al. (61). Driven by the fact that the development of radioresistance is largely determined by factors such as DNA damage response DDR activation after ionizing radiation (IR), cell cycle checkpoints controls, and anti-apoptotic pathways dysregulation, it is essential to investigate the potential of using NEDDylation inhibitors as radiosensitizers (64).

### NEDDylation Inhibitors as Radiosensitizers—DNA Damage Response

One of the major cytotoxic effects of MLN4924 is achieved through the stabilization of its substrate Cdt1, a so-called “DNA replication licensing factor,” which tightly regulates the cell cycle progression by facilitating the formation of the pre-replicative complexes (pre-RC) at the G1 phase of cell cycle (46, 65). To prevent relicensing, which will lead to multiple rounds of DNA replication initiation per cell cycle, Cdt1 is rapidly degraded by the CRL Skp1-cullin1-F-box protein (SCF) right after G1 phase (66) (Figure 2). MLN4924-mediated inhibition of SCF will lead to the accumulation of Cdt1, causing firing of several rounds of DNA replication initiations without cell division, as evidenced by the accumulation of cells with > 4N DNA in flow cytometry (46) (Figure 2). This process will lead to the collision of replication forks and the induction of overwhelming both single- and double-strand DNA damage (67).

The majority of IR-mediated cell killing is mediated by the massive induction of DNA DSBs (64). Radiosensitivity of tumor cells is largely decided by their ability to trigger the DDR, via activation of cell cycle checkpoints and DNA damage repair (64). MLN4924 functions as a radiosensitizer in several types of cancer by potentiating DNA damage and interfering with DDR activation. In the orthotopic xenograft mouse models of human pancreatic cancer and head and neck squamous cell carcinoma (HNSCC), MLN4924 overcame radioresistance via induction of DNA rereplication, leading to prominent induction of DSBs (68, 69). In pancreatic cancer cells, the maximal radiosensitizing effects of MLN4924 was achieved when MLN4924 was administered 24 h prior to receiving RT (69). MLN4924 pretreatment before RT administration will allow time for CRL substrates' accumulation. The radiosensitizing effect of MLN4924 was partially reversed in pancreatic cancer cells with Cdt1 knockdown (69). However, the exact involvement of Cdt1 stabilization in MLN4924-induced radiosensitization needs further investigation.

Expression levels of CRL components were significantly elevated in HNSCC cells compared with those in adjacent normal squamous mucosa of the oral cavity and nasopharynx (68). As a result, DNA rereplication was not observed in the cells of normal tissue (68). Besides HNSCC, hyperactivation of CRLs was also observed in GBM, breast cancer, and liver cancer (70). Therefore, the unique cytotoxic mechanism highlights the potential of NEDDylation inhibitors as radiosensitizers from the perspectives of “*spatial cooperation*,” “*biological cooperation*,” “*normal tissue protection*,” and “*cytotoxic enhancement*” based on the revised Steel framework.

### MLN4924 as a Radiosensitizer—Cell Cycle Arrest

Due to the lethality of unrepaired DNA DSBs, developing new agents to prevent activation of cell cycle checkpoints in response to IR is critical to overcoming radioresistance (71). DDR is initiated by activation of ataxia-telangiectasia mutated (ATM) and ataxia-telangiectasia and RAD3-related (ATR), which will locate the DNA damage and activate various downstream proteins (72). ATM is the major regulator of DDR following IR-induced DSBs, leading to phosphorylation of downstream CHK1 and CHK2 (72). Activated CHKs will then phosphorylate the isoforms of CDC25 phosphatases, triggering their polyubiquitination and degradation (73). Meanwhile, the dephosphorylation and activation of CDK2-cyclinE and CDK1-cyclinB depend on the phosphatase activities of CDC25 (73). As a result, with activation of DDR and subsequent CDC25 degradation, cell cycle arrest will occur at the end of G1 phase or the end of G2 phase to allow time for DNA repair (73) (Figure 2). Among the three isoforms of CDC25s (CDC25A, B, C), CDC25A regulates both early (G1 phase to S phase) and late (G2 phase to M phase) cell cycle checkpoints (Figure 2) (73). Rapid degradation of CDC25A is critical for activating cell cycle arrest upon IR-induced DNA damages (72). The ubiquitination of CDC25A is carried out by the CRL E3 ligase SCF^beta−TrCP^, in which the beta-TrCP (β-transducin repeat-containing protein) facilitates the recruitment of the CDC25A for Ub conjugation (74). MLN4924-mediated inhibition of SCF^beta−TrCP^ will stabilize the CDC25A protein, causing cell cycle checkpoint dysregulation and potentially radiosensitization (Figure 2).

Accumulation of CRL substrates may also induce cell cycle arrest via checkpoint activation. The Wee1 kinase, which phosphorylates and keeps CDK1 in inactive form for activation of cell cycle checkpoints, is another major CRL substrate (Figure 2) (75). Meanwhile, degradation of members of the universal cyclin-dependent kinase inhibitors (CDKIs) family p21 (Cip1) and p27 (Kip1) is also mediated by CRLs (76). As such, several studies reported the activation of cell cycle checkpoints with MLN4924 treatment (77–79). In cell lines of hormone-refractory prostate cancer (HRPC), MLN4924 triggered cell cycle arrest at the G2 phase due to Wee1, p21, and p27 accumulation (79). In the colorectal cancer cell lines of HT-29 and HCT-116, and breast cancer cell lines of SK-BR-3 and MCF7, MLN4924 induced stabilization of p27 and p21, respectively, leading to cell cycle G2/M arrest (77, 78). MLN4924 and RT cotreatment induced a more significant accumulation of Wee1, p21, and p27 than either treatment modality alone, leading to prominent cell cycle arrest and unanimous sensitization all these types of cancer cells toward RT (77–79).

### MLN4924 as a Radiosensitizer—Anti-apoptotic Pathways

Increased radioresistance of cancer cells is developed by the activation of several compensatory pro-survival cell signaling pathways, including phosphatidylinositol 3-kinase (PI3K)/AKT/mTOR pathway, EGFR/mitogen-activated protein kinase (MAPK) pathway and NF-κB signaling pathway (80, 81). The classical theories of how radiation activates these anti-apoptotic pathways state that ionizing events in the cytosol and the mitochondria will generate large quantities of reactive oxygen species (ROS) and reactive nitrogen species (RNS) that will inhibit protein phosphatase (PTPase) activities (81). Radiation can also promote membrane-associated receptor activation by lipid rafts aggregation, leading to activation of downstream pathways (82). Activation of the PI3K/AKT/mTOR, EGFR/MAPK, and NF-κB pathways can facilitate the development of radioresistance by promoting DNA damage repair, and transcriptional upregulation of a myriad of stress-responsive proteins (83, 84) (Figure 2). Therefore, it is critical to understand the impact of NEDDylation inhibition on these compensatory pro-survival pathways activated by RT.

Inhibition of NF-κB pathway is one of the major causes of MLN4924 induced cytotoxicity, as evidenced in the initial studies in acute myeloid leukemia (AML) (49). The inhibitor of nuclear factor kappa B (IκBα) binds to the NF-κB p65 and p50 complex and keeps the heterodimer in the cytosol as an inactive form (85). Activation of the pathway triggers rapid degradation of IκBα via the SCF^β−*TrCP*^ E3 ligase, releasing the p65 and p50 heterodimer for nuclear translocation and transcriptional upregulation of its target genes (86). Treatment of MLN4924 will inhibit the SCF^β−*TrCP*^ and prohibit RT-induced IκBα degradation, with resultant sequestration of p65 and p50 in the cytoplasm (49, 52, 87) (Figure 2). This mechanism is validated in studies showing that eradication of the RING-box protein Rbx2 in the SCF^β−*TrCP*^ complex triggered IκBα stabilization and NF-κB pathway inhibition, leading to re-sensitization of cancer cells toward RT (88) (Figure 2). Furthermore, the existing studies also suggested that the radiosensitizing effect of bortezomib is largely due to the inhibition of the NF-κB pathway (89). As a result, another major radiosensitizing mechanism of MLN4924 is achieved through the NF-κB pathway inhibition (Figure 2).

Several mTOR inhibitors, including everolimus and temsirolimus, are under early Phase (I/II) clinical trials as a radiosensitizer to treat several cancer types such as prostate cancer, GBM, and lung cancer (90–92). In human cancer cell lines of acute lymphoblastic leukemia (ALL), AML, cervical, breast, colon, GBM, and kidney, the activity of mTOR is downregulated by MLN4924 in an almost dose-dependent manner, as evidenced with dephosphorylation of mTOR downstream targets such as p70S6 kinase (51, 93–95). Intrinsic mTOR's upstream inhibitors, including the DEP domain containing MTOR interacting protein (DEPTOR) and the regulated in development and DNA damage responses 1 (REDD1), are substrates for SCF^β*TrCP*^ and cullin4A-RING (CRL4A), respectively. These protein-drug interactions largely explain the unanimous response of mTOR inhibition toward NEDDylation inhibition (50, 51, 55). The significant inhibitory effect of MLN4924 on the PI3K/AKT/mTOR axis has implicated the NEDDylation inhibitors as potential therapeutic radiosensitizers (Figure 2).

In summary, NEDDylation inhibition can block key pro-survival pathways activated with RT via stabilization of their intrinsic upstream inhibitory proteins. The unique role of MLN4924 in blocking these compensatory pathways demonstrated its potential application as a radiosensitizer via “*spatial cooperation*,” “*biological cooperation*,” and “*cytotoxic enhancement*” (4).

## PROteolysis Targeting Chimeras (PROTACs)

**Figure 3:**
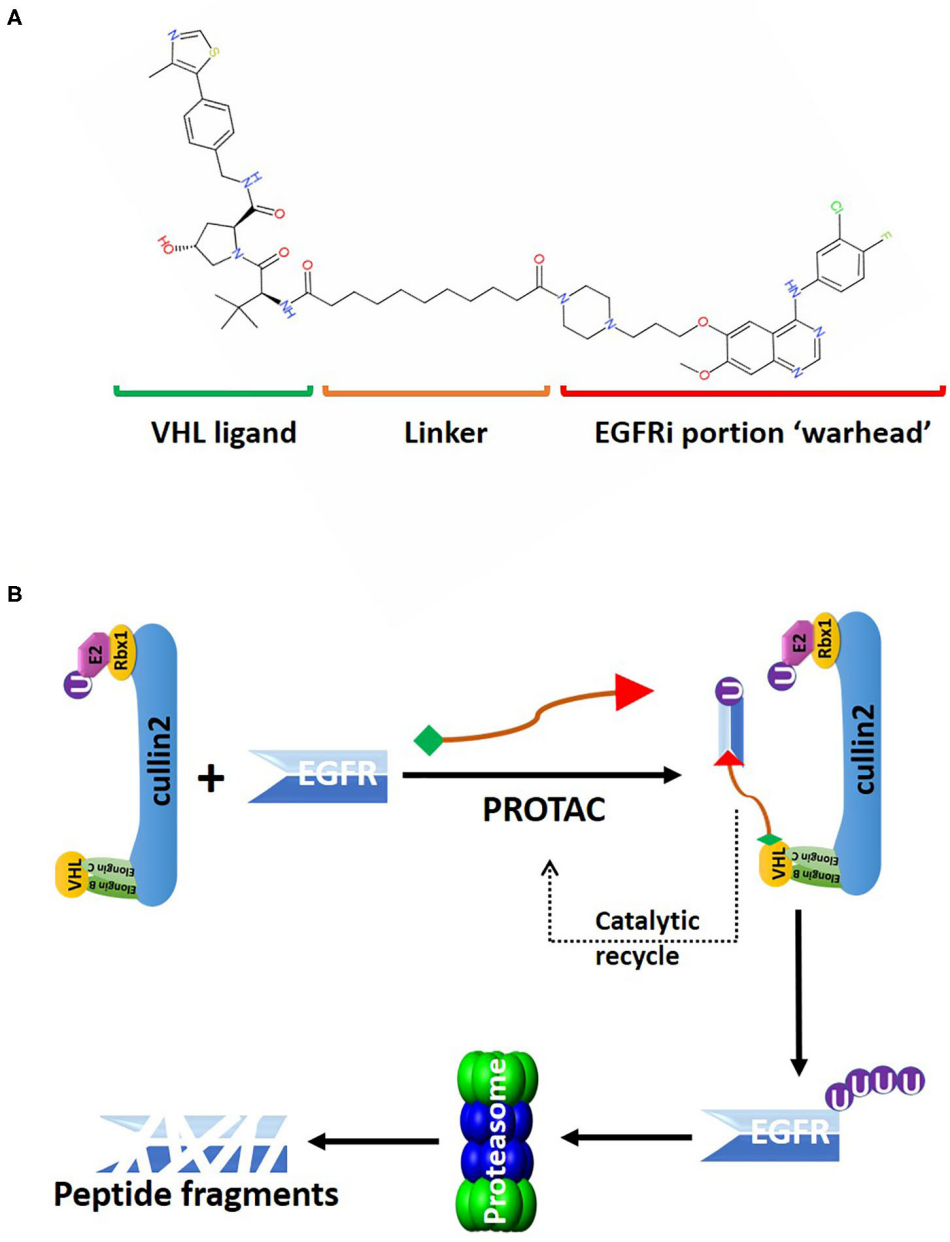
An overview of the PROTAC targeting EGFR for CRL2^VHL^-mediated polyubiquitination. **(A)** 2D structure of the PROTAC MS39 that targets EGFR. The “warhead” potion of the PROTAC is based on EGFR inhibitor (EGFRi) gefitinib. **(B)** MS39 can recruit EGFR for polyubiquitin conjugation by the CRL2^VHL^. MS39 mediated EGFR degradation is a catalytic process, as evidenced with dissociation of the PROTAC from the CRL2^VHL^ complex after EGFR polyubiquitination.

Neither bortezomib nor MLN4924 addresses specific proteins as they broadly inhibit the general machinery necessary for protein degradation. MLN4924 is not selective since all the CRL complexes in the cells are inhibited, blocking the activities of over 400 enzymes (70). The PROteolysis TArgeting Chimeras (PROTACs) technology was developed in recent years to overcome these limitations of targeting the protein degradation machinery (96). PROTACs are heterobifunctional molecules with two different ligands connected via a linker (Figure 3). One end of the PROTAC, i.e., the “warhead,” binds to the protein of interest (POI), and the other end binds to the receptor protein in the CRL complex, thereby promoting the physical interaction of the target protein with the E3 ligase for polyubiquitination (Figure 3) (97). Traditional targeted therapies using occupancy driven pharmacology only affect enzymatic function via competitive inhibition, which requires druggable active sites in those enzymes that are susceptible to mutations and protein overexpression (8). Whereas, polyubiquitinated POIs will be degraded by the proteasome with the eradication of both the enzymatic activities and the scaffold functions of target proteins (97). Furthermore, PROTACs-induced protein degradation is a catalytic process, as PROTACs will dissociate from the CRL complex after POI polyubiquitination and binds to a new target. This unique catalytic property of PROTACs can lead to efficient POI clearance at a very low dose5 (98) (Figure 3).

**Figure 4:**
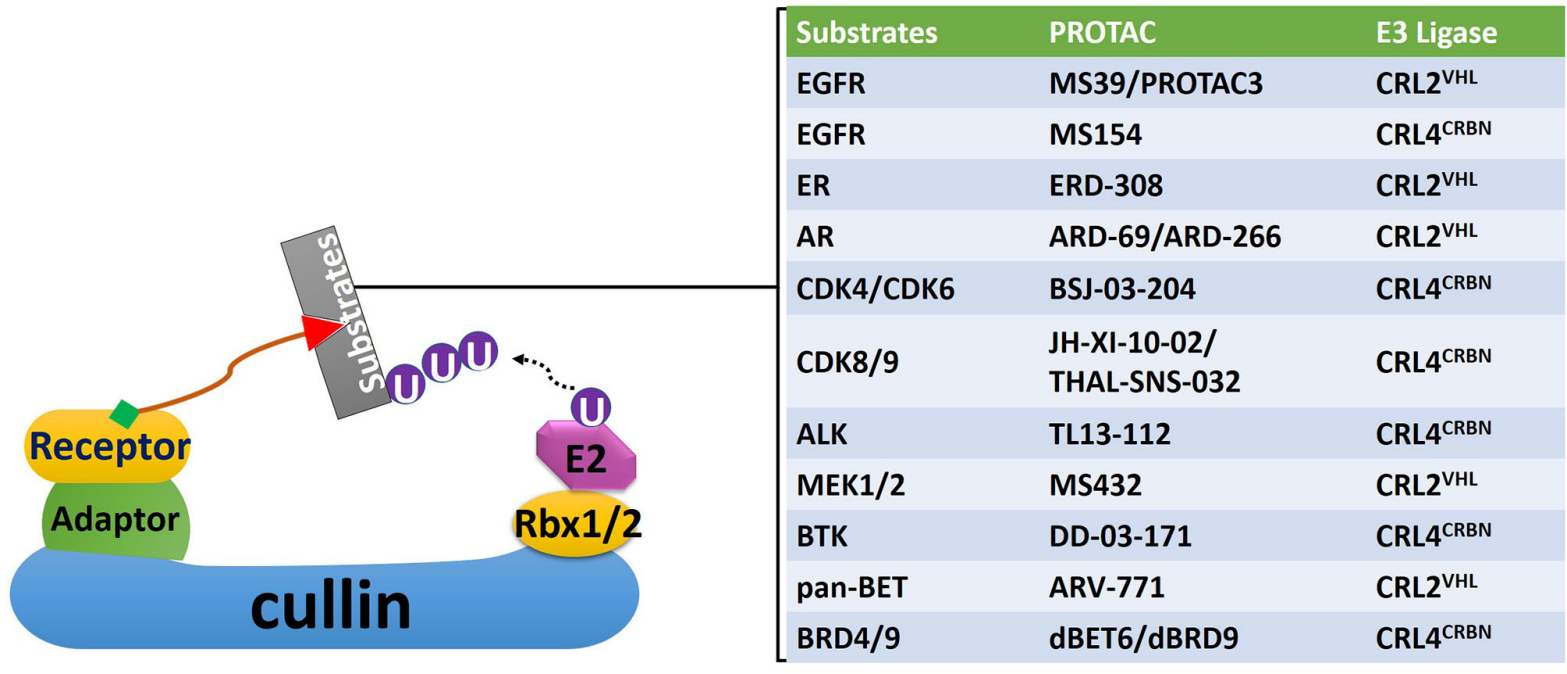
A non-exhaustive list of PROTACs targeting proteins related with the development of radioresistance. Corresponding CRLs that mediate polyubiquitination of these proteins were also identified. EGFR, epidermal growth factor receptor; ER, estrogen receptor; AR, androgen receptor; CDK, cyclin-dependent kinase; ALK, anaplastic lymphoma kinase; MEK, mitogen-activated protein kinase kinase; BTK, Bruton tyrosine kinase; BET, bromodomain and Extra-Terminal motif; BRD, bromodomain.

Due to these unique characteristics, targeting CRL substrates related to the development of radioresistance with PROTACs could provide a new strategy to sensitize cancer cells toward RT (70) (Figure 4). Since 2015, over 30 small-molecule PROTACs have been reported, most of them utilize substrate receptor proteins von Hippel-Lindau (VHL) in the CRL2 complex (CRL2^VHL^), and cereblon (CRBN) in the cullin4-RING complex (CRL4^CRBN^) as the PROTAC binding sites in the E3 ligase (99) (Figure 4). PROTACs against radioresistance-related key substrates of CRL2 and CRL4 have been developed. These substrates include the EGFR, androgen (AR) and estrogen (ER) receptors, CDKs, MAP kinase kinase 1 (MEK1) and MEK2, anaplastic lymphoma kinase (ALK), Bruton tyrosine kinase (BTK), bromodomain and extra-terminal motif (BET) proteins, and bromodomain (BRD) proteins (Figure 4) (100–107). Since all these PROTACs are first-in-class protein degraders developed within the past 5 years, a comprehensive understanding of the underlying molecular mechanism of these novel compounds is essential for developing next-generation radiosensitizers.

### PROTACs as Radiosensitizers—Targeting Receptors

Targeting EGFR using traditional targeted therapies, such as monoclonal antibodies or tyrosine kinase inhibitors (TKIs), as radiosensitizers have gained moderate success in non-small-cell lung cancer (NSCLC), but failed in GBM and HNSCC to improve OS rates (108, 109). Genetic alterations, including amplification, rearrangement, altered splicing, and mutations, that regulate *EGFR* expression levels and protein activities in GBM and HNSCC, will eventually lead to the development of resistance toward EGFR inhibitors (108, 110). Lysosome-independent degradation of EGFR is mediated by CRL2^VHL^, consistent with the report in clinical trial showing stabilization of EGFR with bortezomib-mediated proteasome inhibition (32, 111). Recently, several PROTACs were developed to target EGFR for CRL2^VHL^-mediated degradation. PROTAC-based technology offers great flexibility in choosing the clinically relevant forms of EGFR proteins targeted for degradation by changing the “warhead” of the degrader (Figure 3). For example, the lapatinib-based PROTAC largely degraded the wildtype, and exon-20 insertion mutant forms of EGFR; the gefitinib-based PROTAC selectively degraded EGFR with exon-19 deletion, and L858R point mutation; afatinib-based PROTAC degraded double mutant (L858R/T790M) EGFR (112). All these PROTACs can efficiently eliminate EGFR at low-nanomolar concentrations, and exerted sustained inhibitory effects on cancer cell proliferation and downstream kinases signaling of EGFR (112).

RT-induced overexpression of hormonal receptors, including AR and ER, plays a vital role in mediating radioresistance in prostate and breast cancers, respectively (113, 114). In castration-resistant prostate cancer (CRPC) cells, the PROTAC ARD-61 can efficiently degrade AR and inhibited cancer cell proliferation with half-maximum inhibitory concentration (IC:50) values <500 nM, regardless of AR mutations, and expression status of AR splice variants, such as AR splice variant-7 (AR-V7) (102). Meanwhile, the viability of cells not expressing AR was not affected (102). Another AR degrader ARD-69 has DC50 values of <1 nM in prostate cancer cell lines LNCaP and VCaP (DC50: the concentration at which 50% of the target protein has been degraded) (115). The importance of these PROTACs as potential radiosensitizers for prostate cancer is emphasized by the study showing that targeted degradation of RT-increased AR with FDA-approved AR degradation enhancer, dimethylcurcumin (ASC-J9), significantly sensitized prostate cancer toward radiation in xenograft models, while conventional anti-androgen drugs, such as enzalutamide, has no radiosensitizing effects (114). Meanwhile, the ER degrader ERD-308 can induce over 95% of ER degradation at concentrations of <5 nM in ER+ breast cancer cell lines of MCF-7 and T47D (101). Given the synergistic effect of typical anti-estrogenic drugs with RT in the breast, and cervical cancers, these ER degraders can also be used as potential radiosensitizers alongside with RT (116). More importantly, these degraders can overcome the common resistant mechanism developed during anti-hormonal therapy.

### PROTACs as Radiosensitizers—Targeting Oncogenic Kinases

It is well-known that many patients will eventually become drug resistant and develop disease relapse with prolonged treatment of protein kinase inhibitors (117). The mechanism of drug resistance is mainly attributed to the kinome rewiring effect, whereby reactivation of the oncogenic pathways restored via compensatory feedback activation of alternative kinases (117). Particularly, RT-induced activation of MEK1/2 is associated with radioresistance is found in several human malignancies (118, 119). Targeted inhibition of MEK1/2 using kinase inhibitors led to radiosensitization in several types of cancer, such as astrocytoma and pancreatic tumor (120, 121). Meanwhile, CDK inhibitors, especially those against CDK4 and CDK6, synergized with RT in killing GBM cells and prolonged survival in the orthotopic GBM model (122). However, resistance toward MEK1/2 and CDK inhibitors will inevitably occur with prolonged treatment (123).

Treatment with PROTACs will lead to catalytic degradation of specific kinases, offering sustained inhibition on their downstream targets (112). PROTACs for kinases critical for radioresistance, including CDKs, MEK1/2, ALK, and BTK, have been recently developed (103, 105–107). Given their unique pharmacological characteristics, these PROTACs carry great potential as radiosensitizers. For example, the PROTAC MS432 recruits MEK1/2 for CRL2^VHL^-mediated polyubiquitination (105). It can suppress extracellular signal-regulated kinase (ERK) phosphorylation and efficiently inhibit colorectal cancer cell proliferation with DC50 values of 31 nM and 17 nM for MEK1 and MEK2 in HT-29 cells, respectively (105). Meanwhile, the CDK6 degrader was recently developed by linking the FDA-approved CDK6 inhibitor palbociclib with a thalidomide derivative for targeted CDK6 polyubiquitination by CRL4^CRBN^ (124). Given that the combination treatment of RT with CDK inhibitors palbociclib and ribociclib are well-tolerated in malignancies such as breast cancer and glioma (NCT 02607124), CDKs-targeting PROTACs are great pharmaceutical candidates for radiosensitizers (125).

## Conclusion and Discussion

In summary, protein turnover is a dynamically regulated process influencing many important biological functions, including DDR, cell cycling, and signaling transductions. Pharmacological intervention of protein turnover offers a new therapeutic window for radiosensitization. Driven by their unique cytotoxic mechanisms, the novel strategies targeting the UPS with NEDDylation inhibitors and the PROTACs carry great potential as radiosensitizers to improve the efficacy of RT. The NEDDylation inhibitor MLN4924 exerts several cytotoxic functions, including DNA damage, cell cycle checkpoints dysregulation, and inhibition of NF-κB, and mTOR pathways (Figure 2). Preclinical studies had validated the efficacy of NEDDylation inhibitors as radiosensitizers. Meanwhile, recent progress in PROTAC technology has shown significant improvements in terms of the cellular permeability and substrate specificity. The PROTACs can selectively recruit key proteins related to radioresistance, such as EGFR, AR, ER, MEK1/2, and CDKs, for CRL-mediated polyubiquitin conjugation and subsequent degradation. Based on these strong basic and preclinical investigations hereby summarized, further clinical studies using NEDDylation inhibitors and PROTACs as radiosensitizers are warranted for the therapeutic gain of human malignancies.

## Author Contributions

SZ wrote the original manuscript. WT edited and wrote the manuscript. All authors contributed to the article and approved the submitted version.

## Conflict of Interest

The authors declare that the research was conducted in the absence of any commercial or financial relationships that could be construed as a potential conflict of interest.
